# Factors Affecting Domestic Water Consumption in Rural Households upon Access to Improved Water Supply: Insights from the Wei River Basin, China

**DOI:** 10.1371/journal.pone.0071977

**Published:** 2013-08-16

**Authors:** Liangxin Fan, Guobin Liu, Fei Wang, Violette Geissen, Coen J. Ritsema

**Affiliations:** 1 State Key Laboratory of Soil Erosion and Dryland Farming on the Loess Plateau, College of Natural Resources and Environment, Northwest A&F University, Yangling, China; 2 School of Surveying and Land Information Engineering, Henan Polytechnic University, Jiaozuo, China; 3 Institute of Soil and Water Conservation, Chinese Academy of Sciences and Ministry of Education, Yangling, China; 4 Soil Science Centre, Alterra, Wageningen University and Research Centre, Wageningen, The Netherlands; University of Florida, United States of America

## Abstract

Comprehensively understanding water consumption behavior is necessary to design efficient and effective water use strategies. Despite global efforts to identify the factors that affect domestic water consumption, those related to domestic water use in rural regions have not been sufficiently studied, particularly in villages that have gained access to improved water supply. To address this gap, we investigated 247 households in eight villages in the Wei River Basin where three types of improved water supply systems are implemented. Results show that domestic water consumption in liters per capita per day was significantly correlated with water supply pattern and vegetable garden area, and significantly negatively correlated with family size and age of household head. Traditional hygiene habits, use of water appliances, and preference for vegetable gardening remain dominant behaviors in the villages with access to improved water supply. Future studies on rural domestic water consumption should pay more attention to user lifestyles (water appliance usage habits, outdoor water use) and cultural backgrounds (age, education).

## Introduction

Water is the most important natural resource for sustainable development and quality of life, yet it is unevenly distributed; almost one-fifth of the world’s population lives in regions where water is scarce and one-quarter suffer from severe water shortage [1]. To resolve these problems, WHO, UNICEF, and other international organizations have exerted tremendous effort into ensuring secure domestic water supply around the world. Consequently, the populations provided access to improved water supply increased from 76% in 1990 to more than 89% in 2010 globally, accounting for 90% or more of the populations of Latin America, Northern Africa, and large parts of Asia, as well as 61% of the population of sub-Saharan Africa [1], [2]. In developing countries, the rural populations that are afforded access to safe water supply have considerably increased from 36% in 1990 to 56% in 2010 [3]. Despite the progress achieved, however, improved domestic water supply systems are expected to continue being affected by water shortage–a problem that will worsen because of the population increase, economic growth, improved living conditions, and lifestyle changes in rural areas [4]. An urgent requirement, therefore, is the development of effective strategies and public policies on water management in villages with access to improved water supply.

A clear understanding of water use patterns and the factors that affect water consumption is critical to the effective management of water supply and effective design of related public policies. Water use patterns are highly complex processes that are influenced by many factors, including seasonal variability and water availability [5], [6], water supply restrictions [7], tariff structure and pricing [8], household characteristics [4], [9], and attitudes and intentions regarding water conservation [10]. These factors both directly and indirectly drive water consumption and usage behaviors [11]. In investigating the aforementioned issues, researchers have focused on water demand in urban regions. Demand in rural households of developing countries, where traditional and cultural influence on water consumption is expected, has not been sufficiently studied [6].

According to the most recent data from the World Bank, almost half of the world’s population (3.4 billion, 49.3% of the total) lives in rural regions. Of these, 76.5% (2.6 billion) live in developing countries and 21.7% (0.74 billion) live in China [3]. In rural areas of the Wei River Basin, local people’s lifestyles have considerably improved, as evidenced by the annual household income growth rate of more than 10% and the popularization of bathrooms, washing machines, and solar water heaters. These improvements are a result of the Chinese Western Region Development Strategy for sustainable development implemented in 1999. In addition, the proportion of the basin’s rural population gaining access to improved water supply in 2010 amounted to 87%, a significant increase over 45% in the previous decade [12]. The government should fully consider the aforementioned findings as it formulates public policies on water management. To provide basis for sound policy making, we examined the key factors that affect the domestic water use patterns and water consumption in the Wei River Basin, with the aim of identifying effective strategies for ensuring water supply security and effective water supply management in rural areas of developing countries.

Our analyses were guided by two questions:

1. How do improved water supply systems affect household water use behaviors and consumption?
2. In rural areas of developing countries, what are the key factors that affect domestic water consumption and water use behavior once households gain access to improved water supply?

## Materials and Methods

### Description of Study Area

**Figure 1:**
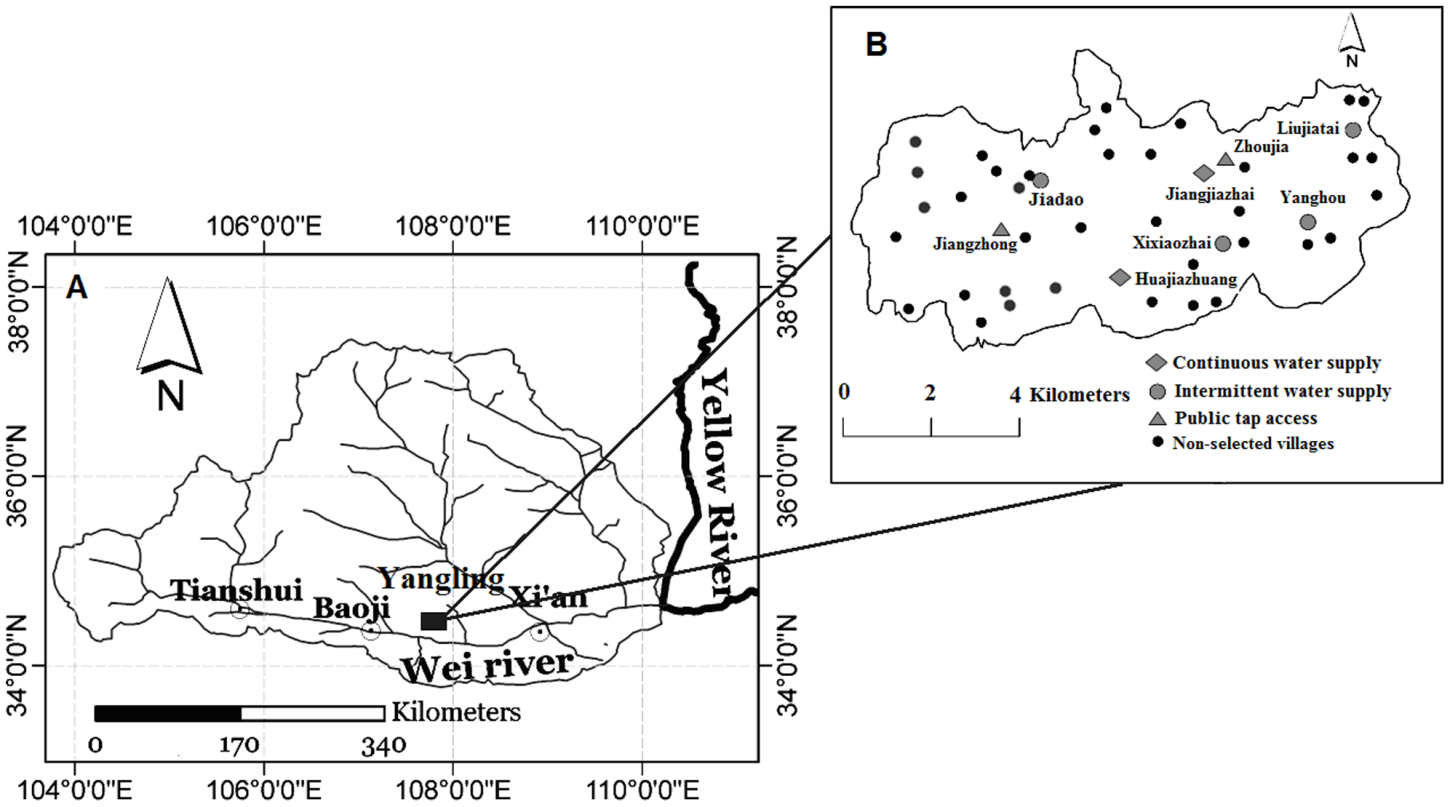
Location of the study region. Note: A and B represent the locations of the Wei River Basin and Yangling district, as well as the sampled villages, respectively.

The study was carried out in the Yangling district (34°17′N, 107°57′E –34°20′N, 108°04′E, 500–600 m elevation, 35 km^2^) in the central region of the Wei River Basin, 74 km west of Xi’an city, NW China (Fig. 1A). This region has a semi-humid climate, with a mean annual temperature of 12.9°C and a frost-free period of 213 days. The average annual precipitation is 635 mm and the annual evaporation is 1200 mm. The topography is flat; 84% of the land is cropland, with wheat and corn being the main crops [13]. A population of 14,300 live in the 45 villages scattered throughout the region. Groundwater is the main water resource, supplying 73% of the water for irrigation and all forms of domestic use. Forty-two villages (5 with continuous water supply and 37 with intermittent water supply) obtain water via the pipelines from 19 water supply utilities across the region. Each utility has a water tank (volume range: 8–15 m^3^), a well (depth: 25–40 m), and a pumping station. Groundwater is pumped to the tank, from which pipelines supply water to households. The remaining three villages do not have pipelines, and residents carry water from public taps for domestic use.

### Ethics Statement

We obtained ethical approval from the Academic Committee of the Institute of Soil and Water Conservation, Chinese Academy of Sciences. All the investigations were approved and supervised by local authorities of the Water Resources Bureau of Yangling, China. We informed respondents that participation in the survey was entirely voluntary and that that they were free to deny us information at any time without providing justification. Written informed consent for data collection was obtained before questionnaire administration, after which the data collected were used in analyses.

### Questionnaire Survey and Data Collection

The survey was carried out from June to November 2010. During the first stage (June 2010), local village leaders were interviewed for information on the socioeconomic situation, water supply systems, and water prices in each village. A pre-survey was conducted in 20 households to better understand the water use patterns and behaviors of local residents. During the second stage (July–November 2010), interviews were conducted in 247 randomly selected households from eight villages where three water supply systems are implemented: continuous water supply (74 households, 2 villages), intermittent water supply (104 households, 4 villages), and public tap access (69 households, 2 villages) (Fig. 1B and Table 1). One family member older than 18 years from each household was interviewed; 165 (66.5%) of the interviewees were heads of their households. The questionnaire items were based on the results of the pre-survey:

**Table 1:** Demographic characteristics of the sampled villages.

Water supply types	Villages	Sampled households	Annual household income (US $)	Net family size	Water supply time (h/d)	Water prices (US $/m^3^ )
Public tap access	Zhoujia, Jiangzhong	69	4580	3.0	2.0	0.32 and 0.24
Intermittent water supply	Yanghou, Xixiaozhai, Jiadao, Liujiatai	104	4660	3.1	6.0–2.0	0.16–0.32
Continuous water supply	Jiangjiazhai, Huajiazhuang	74	4810	2.8	24.0	0.24

Net family size: excludes members residing outside a household for more than eight months.

1. characteristics of the head of household (age, gender, and educational level) [11], [14] and socioeconomic situation (net family size, household income, vegetable garden and yard area, and possession of livestock, washing machines, and solar water heaters) [9], [15], [16];
2. domestic water consumption (indoor consumption includes water used for drinking, personal hygiene (i.e., washing face, hands, and feet), kitchen activities, showering and laundry; outdoor consumption includes water used for vegetable garden, livestock needs, house and yard cleaning) [17], [18];
3. dominant water use behaviors (indoors: frequency of laundry, frequency of personal hygiene activities, water appliance usage ratio, etc; outdoors: frequency of vegetable garden watering, and frequency of house and yard cleaning) [14], [19].

### Data Analyses

The results were statistically analyzed using SPSS 15.0 and EViews 7.0 [20], [21]; the analyses provided the mean ± standard deviation for each case. We compared water consumption patterns (total indoor and outdoor usage: personal hygiene, drinking, kitchen activities, showering, laundry, vegetable gardening, livestock raising, and house and yard cleaning), dominant water use activities (personal hygiene, laundry, showering and vegetable garden watering) across the three water supply systems. The data were analyzed by one-way ANOVA followed by Tukey’s post hoc test for multiple comparisons. Differences at a p-value <0.05 were considered statistically significant. Simple correlation coefficient and stepwise regression analyses were performed to identify the key factors that affect domestic water consumption. Due to the key factors for water consumption may not exhibit causal relationships, and the interactions among variables may affect outcomes to an extent greater than the individual contribution of each variable [22], [23]. Thus, we combined Granger causality analysis with path analysis to determine the relationships among the key factors. The Granger causality test was conducted using EViews 7.0. Path analysis is a method of separating direct and indirect effects and measuring the relative importance of involved causal factors [24], [25]. For this reason, similar to the Fourier amplitude sensitivity test used in sensitivity analysis [26], [27], this method is widely used in research on social and behavioral phenomena [28], [29], such as energy consumption [30], drug use [31], nutrient intake [32], and alcohol consumption [33].

## Results

### Profiles of Chinese Water and Sanitation Programs

**Figure 2:**
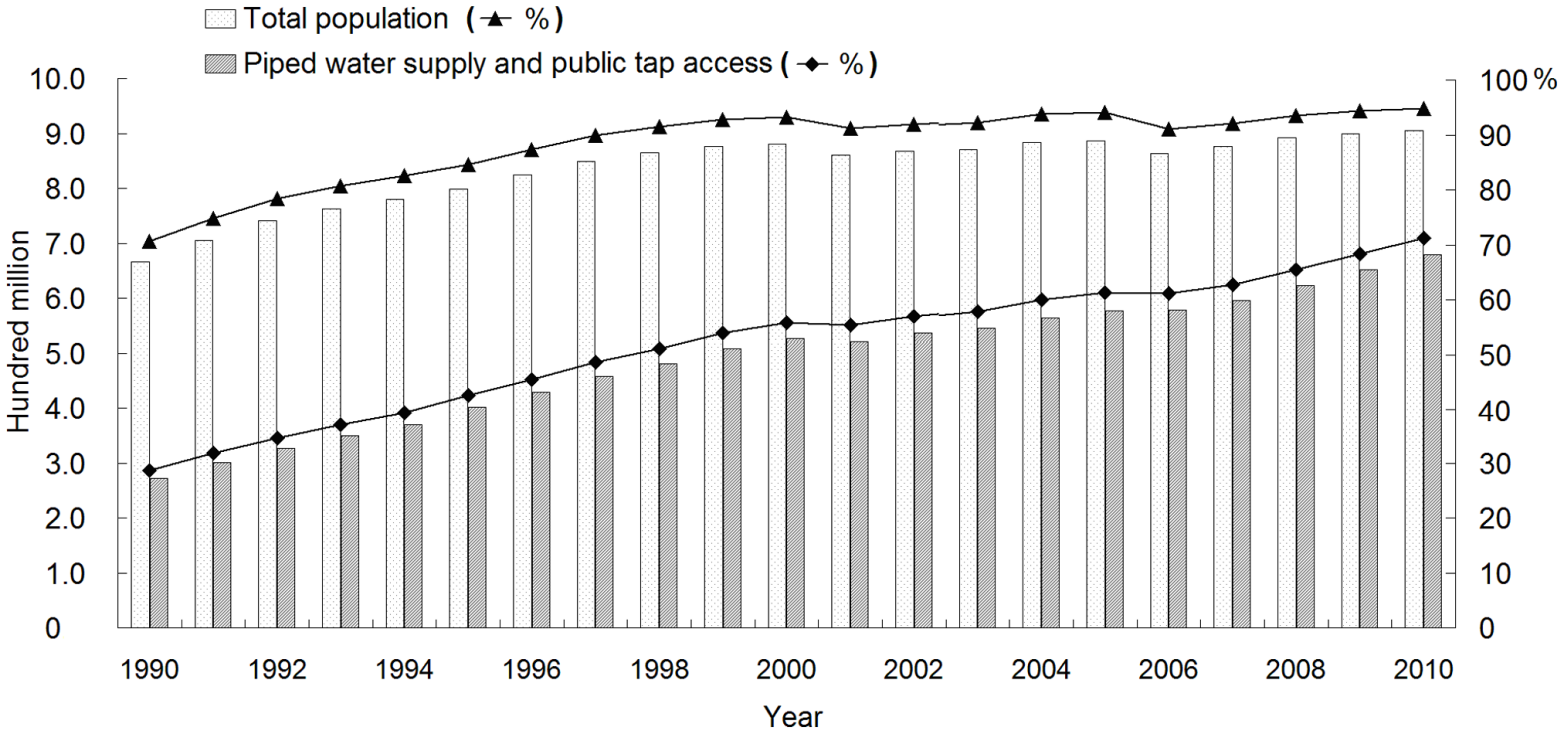
Total population benefitting from improved drinking water supply in rural China. Note: Data source: [12], [48].

Given the efforts of the Chinese government to improve the safety of drinking water supply since the 1960s (Table 2), household access to improved water supply and sources has significantly increased in rural China, particularly over the past two decades. The population received piped water supply and access to public taps increased from 271 million in 1990 to 682 million in 2010 (Fig. 2). In the Millennium Development Goals Report, an estimated population of more than 457 million in China has gained access to improved water supply since 1990 [1]. Water supply in rural China has recently undergone massive transition to safer systems with the implementation of the Mother Cisterns Project (2001–2011) and Chinese Safe Drinking Water Project (2005–2015), which aim to provide safe drinking water to all rural residents before 2015 [34]. As part of these projects, thousands of small-scale water utilities have been established in rural villages in the Wei River Basin. The water supply systems in the region are continuous piped water supply, intermittent piped water supply, and public tap access, which provide for the water consumption needs of more than 57% of the rural population. Village committees in local areas are responsible for formulating water supply strategies and implementing daily management; the water prices of these utilities varying from US$0.16/m^3^ to US$0.32/m^3^, as indicated in the survey.

**Table 2:** Efforts to improve rural drinking water supply in China.

Period	Measures
1950s	The government incorporated irrigation engineering into water management schemes to solve drinking water issues.
1960s	(1) Rural drinking water supply projects were planned.(2) People were organized to dig water cellars, wells, and water pools.
1970s	(1) Machinery wells were widely developed to improve water supply.(2) By 1979, about 40 million people and 21 million livestock had access to improved drinking water supply.
1980 to 1990	(1) The rural drinking water supply plan was implemented beginning 1983.(2) World Bank low-interest loans were used to support rural water supply projects (1984–1989).(3) By 1990, 2.2 million rural water supply projects were developed, and 132 million people and 78.87 million livestock had access to improved drinking water supply.
1990 to 2000	(1) “The Ten-Year Plan” and “Eighth Five-Year Plan” for Rural Drinking Water Supply were issued in 1991.(2) The “Eight-seven Poverty Reduction Plan” was implemented for improved rural drinking water supply in 1994.
2000 to 2010	(1) The Mother Cisterns Project (2001–2011) was implemented in 2001.(2) The Chinese Safe Drinking Water Project (2005–2015) was implemented in 2005.

Data sources: [34], [48].

### Water Consumption and User Behavior Under Improved Water Supply

**Figure 3:**
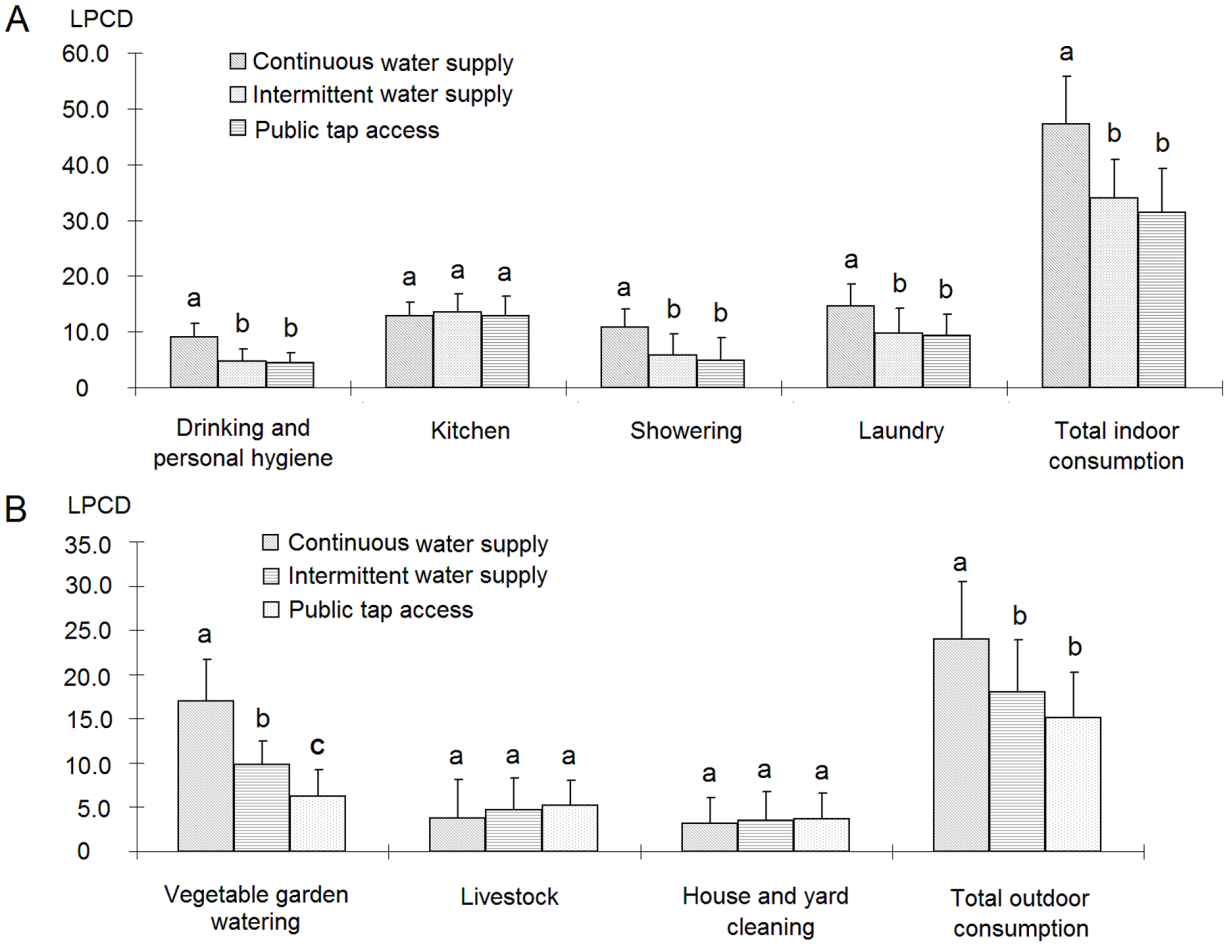
Water consumption (liters per household per day) (means and SD) under different water supply systems. n = 74 for continuous water supply, n = 104 for intermittent water supply, and n = 69 for public tap access. Note: A and B represent the indoor and outdoor water consumption of the sampled villages, respectively. Significant differences in activities existed among different water supply systems (p<0.05), as indicated by the Tukey’s post hoc tests (a>b>c).

The average levels of water consumption for domestic use were 71.3 liters per capita per day (Lpcd), 52.0 Lpcd, and 46.5 Lpcd for villages with continuous piped water supply, intermittent piped water supply, and public tap access, respectively. The villages with continuous water supply and those with the two other supply systems significantly differed in terms of water consumption for laundry, showering, personal hygiene, and vegetable gardening. The villages with intermittent water supply and public tap access showed no significant difference in water consumption patterns, except for watering vegetable gardens (Fig. 3). The water supply patterns affected water consumption through indoor and outdoor water usage. The villages with continuous piped water supply exhibited more frequent water usage for personal hygiene, water appliances (washing machine and solar water heater), and vegetable gardens than did the villages with either intermittent piped water supply or public tap access. The water supply patterns significantly affected the traditional practice of sharing water among family members for hands, feet, and face washing; the households in the villages with continuous piped water supply engaged in less frequent water sharing than did those in the villages with the two other water supply systems (Table 3). Despite the significant changes in water use behaviors because of improved water supply, traditional practices (i.e., sharing water and using a basin for body scrubbing), frequency of showers, low usage of water appliances, and preference for vegetable gardening remained dominant, even in the villages with continuous water supply.

**Table 3:** Traditional behaviors related household water usages (Mean ± SD).

Habits associated with water usage	Continuous watersupply (n = 74)	Intermittent water supply (n = 104)	Public tap access (n = 69)
*Personal hygiene*			
Face, hands, and feet washing/person	36.3±12.0^a^	22.9±9.6^b^	24. 5±12.3^b^
Sharing water for washing hands, feet, and face (ratio: cases of shared waterwhile washing hands, feet, and face/total hands, feet, and face washing)**/**person	0.20±0.13^b^	0.57±0.20^a^	0.54±0.19^a^
*Laundry*			
Use of washing machine of household with a washing machine (ratio: washingmachine use/total washing)/household	0.20±0.12^a^	0.12±0.1^b^	0.16±0.12^b^
*Showering*			
Frequency of showers/person	2.9±0.84^a^	2.6±0.82^b^	2.5±0.86^b^
Ratio of basin use during body scrubbing (ratio: basin use during bodyscrubbing/total shower frequency)/household	0.40±0.09^a^	0.41±0.09^a^	0.42±0.11^a^
Use of solar water heaters during showers in a household with solar water heaters(ratio: shower with the aid of a solar water heater/total shower frequency)/household	0. 92±0.06^a^	0. 90±0.06^a^	0. 77±0.07^b^
*Vegetable garden watering, and house and yard cleaning*			
Frequency of vegetable garden watering/household	2.9±1.3^a^	1.4±0.7^b^	1.9±1.0^b^
Frequency of house and yard cleaning/household	1.6±0.9^a^	1.4±0.9^a^	1.4±1.1^a^

Means with different superscripts within each row differ significantly (P<0.05) a>b>c.

### Factors Affecting Domestic Water Consumption

**Figure 4:**
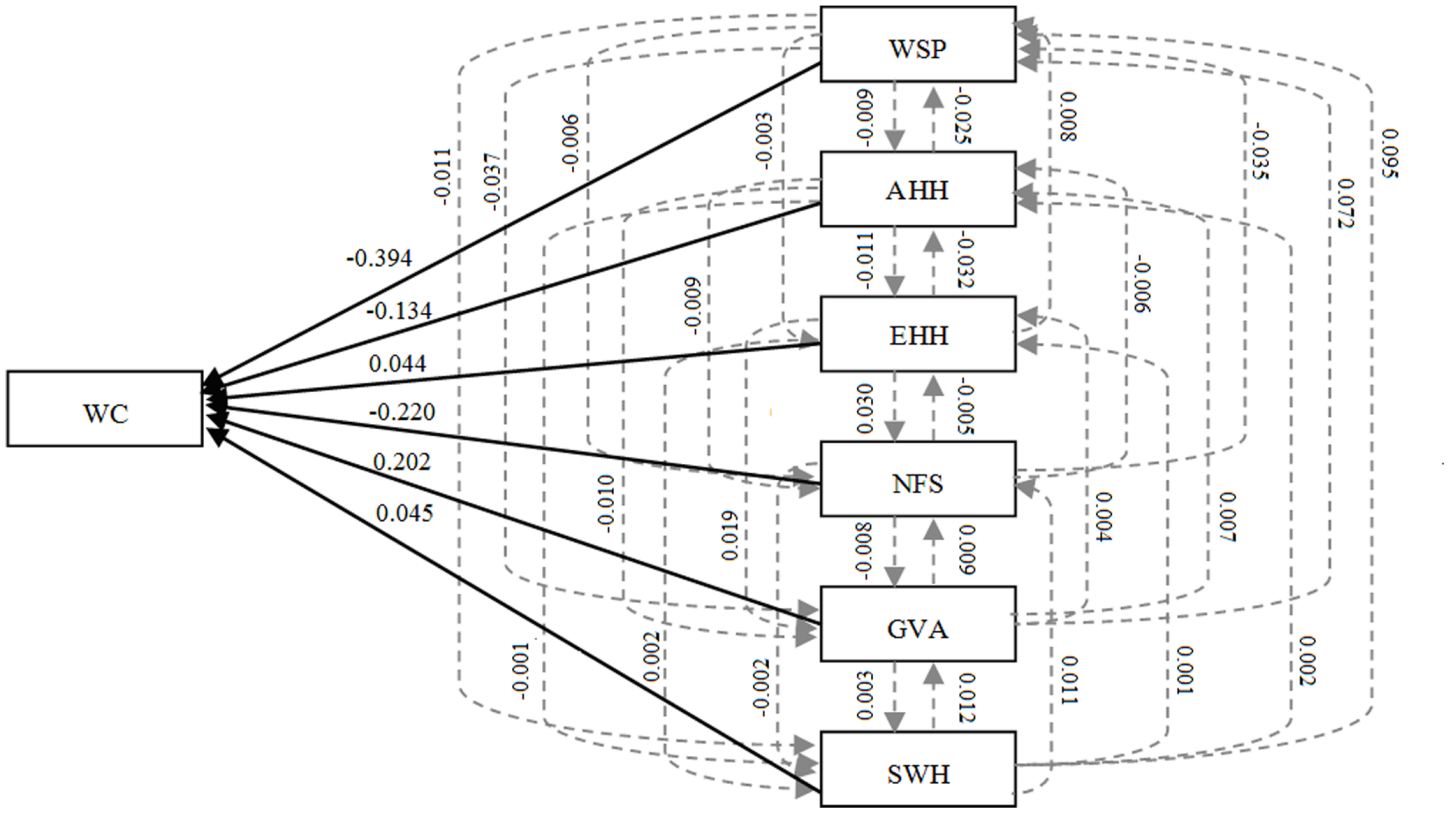
Path analysis results on the effects of six key factors on water consumption. Note: The solid arrows denote direct effects and the dotted arrows denote indirect effects. The numbers indicate the coefficients of correlation between the two variables joined by an arrow. The variable at the base of the arrow is the independent variable.

We performed simple correlation analysis to examine 13 variables that potentially affect water consumption in rural households (Table 4). The variable most strongly associated with Lpcd was water supply pattern (WSP) (r = –0.487, p<0.01), followed by vegetable garden area (VGA) (r = 0.314, p<0.01), net family size (NFS) (r = –0.278, p<0.01), age of household head (AHH) (r = –0.223, p<0.01), household income (HI) (r = 0.191, p<0.01), solar water heater (SWH) (r = 0.173, p<0.01), and educational attainment of household head (EHH) (r = 0.144, p<0.05). NFS and AHH was negatively correlation with Lpcd. An older family member or a member of a huge family usually consumes less water than does a young family member or a member of a small family. The Granger causality analysis indicates that WPS, VGA, NFS, AHH, SWH, and EHH exerted strong directional causal effects on WC; no Granger causality was found between HI and WC, suggesting that HI does not have a causal relationship with WC (Table 5). The effects of the key factors on WC can be classified into direct and indirect effect coefficients by path analysis. Only WPS (direct path coefficient (DPC) = –0.394), NFS (DPC = –0.220), VGA (DPC = 0.202), and AHH (DPC = –0.134) exerted strong direct effects on WC (Fig. 4). The multiple regression analysis shows that WSP, NFS, VGA, and AHH significantly contributed to the prediction of Lpcd and explained 37.1% of the information on variations in Lpcd; EHH and SWH which did not significantly contribute to the prediction, were excluded (Table 6). EHH (DPC = 0.044) and SWH (DPC = 0.045) imposed largely indirect effects on WC, in which the effects of EHH occurred primarily via AHH (indirect path coefficient (IDPC) = –0.032) and NFS (IDPC = 0.030), and those of SWH occurred mainly through WSP (IDPC = 0.095) (Fig. 4).

**Table 4:** Relationship between water consumption and the 13 variables (n = 247).

	WC	WSP	WP	AHH	EHH	HHS	CN	HI	NFS	VGA	YA	LN	WM	SWH
Water consumption (WC)/Lpcd	1													
Water supply pattern (WSP)	−0.487 **	1												
Water price(WP)/US$/m^3^	0.118	−0.115	1											
Age of household head (AHH)/year	−0.223 **	0.064	−0.067	1										
Educational attainment of household head (EHH)/year	0.144 *	−0.021	0.004	−0.239**	1									
Household head sex(HHS)	−0.054	−0.043	0.020	0.001	0.053	1								
Children no. (CN)/household	−0.034	0.087	−0.115	−0.086	0.006	−0.020	1							
Household income (HI)/US$/household	0.191 **	−0.103	0.007	−0.411**	0.038	0.003	0.102	1						
Net family size(NFS)/household	−0.278 **	0.028	0.012	0.042	−0.122	0.152*	−0.053	−0.006	1					
Vegetable garden area (VGA)/household	0.314 **	−0.182**	0.060	−0.049	0.094	−0.029	−0.059	0.048	−0.041	1				
Yard area(YA)/household	0.002	−0.050	−0.076	0.090	−0.115	−0.037	−0.028	−0.040	−0.034	0.016	1			
Livestock no. (LN)/household	0.066	0.007	0.026	0.036	0.008	−0.041	0.066	−0.094	−0.003	0.091	0.070	1		
Washing machine(WM)/household	−0.038	0.018	−0.018	−0.101	0.135*	0.000	−0.027	0.107	−0.033	−0.081	−0.012	−0.101	1	
Solar water heater(SWH)/household	0.173 **	−0.241**	0.027	−0.014	0.009	−0.032	0.011	0.060	−0.049	0.057	−0.016	0.011	−0.043	1

* Probability <0.05;

** <0.01.

WSP: 1, 2, and 3 represent continuous piped water supply, intermittent piped water supply, and public tap access, respectively.

NFS: excludes members residing outside a household for more than eight months.

HHS: men = 1, women = 2.

**Table 5:** Results of Granger causality test on the key variables.

Variables	Null Hypothesis	F-value	P- value	Variables	Null Hypothesis	F-value	P- value
WSP - WC	WSP ≠> WC	24.588**	0.000	AHH - WC	AHH ≠> WC	4.895**	0.008
	WC ≠> WSP	0.366	0.694		WC ≠> AHH	1.223	0.296
EHH - WC	EHH ≠> WC	4.131*	0.017	HI - WC	HI ≠> WC	0.517	0.597
	WC ≠> EHH	2.101	0.125		WC ≠> HI	1.798	0.168
NFS - WC	NFS ≠> WC	6.806**	0.009	VGA - WC	VGA ≠> WC	4.592**	0.004
	WC ≠> NFS	1.502	0.225		WC ≠> VGA	0.459	0.711
SWH - WC	SWH ≠> WC	7.609**	0.006	WSP - VGA	WSP ≠> VGA	10.867**	0.001
	WC ≠> SWH	1.043	0.354		VGA ≠> WSP	0.008	0.992
WSP - SWH	WSP ≠> SWH	3.914*	0.021	AHH - EHH	AHH ≠> EHH	3.716*	0.026
	SWH ≠> WSP	0.199	0.820		EHH ≠> AHH	1.274	0.282
AHH - HI	AHH ≠> HI	16.481**	0.000	EHH - WM	EHH ≠> WM	5.568*	0.019
	HI ≠> AHH	1.168	0.313		WM ≠> EHH	0.857	0.426
HHS - NFS	HHS ≠> NFS	2.340	0.099				
	NFS ≠> HHS	0.054	0.948				

* P<0.05;

** <0.01.

**Table 6:** Stepwise regression of the 6 independent variables (WSP, VGA, EHH, SWH, NFS and AHH) significantly correlated with Lpcd (n = 247).

Step n	Variableentered	MultipleR	R^2^	AdjustedR^2^	F	Sig.
1	WSP	0.487	0.237	0.234	75.648	.000
2	NFS	0.541	0.292	0.287	50.008	.000
3	VGA	0.584	0.341	0.333	41.623	.000
4	AHH	0.609	0.371	0.361	35.463	.001

EHH and SWH did not significantly contribute to the prediction, were excluded from regression.

WSP: 1, 2, and 3 represent continuous piped water supply, intermittent piped water supply, and public tap access, respectively.

NFS: excludes members residing outside a household for more than eight months.

## Discussion

Piped water supply modes (continuous and intermittent water supply) and public tap access are the main types of improved water supply systems, supplying water to 973 and 260 million rural residents worldwide, respectively [2]. Encouraged by low investments, easy management, and effective control of water demand; intermittent water supply systems and public tap access are prevalently used in developing countries [35]. We found that water consumption and usage significantly varied among the three water supply systems; the households with intermittent water supply or public tap access consumed less water than did those with continuous water supply. We also found that intermittent water supply and public tap access introduced hygiene risks by exposing the residents to water-borne (stored water) diseases [36], [37] and by encouraging the traditional practice of sharing water for hygienic purposes given the inconvenience of fetching water from taps (Table 3).

Vegetable gardening is crucial to rural households in developing countries, as confirmed by Chadha and Oluoch [38]. It increases the annual income of small farm families by approximately 30% through providing fresh vegetables and reducing the food budget. Our results show that vegetable gardening strongly affected water consumption because watering gardens accounted for the largest outdoor water use, or specifically, more than 50% of outdoor water consumption. Economic concerns are the main reasons why residents are unwilling to decrease planted areas and watering frequency.

Numerous studies have shown a strong correlation between the age of household head and net family size and water consumption [5], [9]. Keshavarzi et al. [14] reported that the low level of education of elders regarding environmental matters lead them to consume more water than do younger people. By contrast, the present study shows that older people tended to use less water because of traditional practices of water usage (washing hands, showering, and sharing water among family members) and their unfamiliarity with water appliances. Changing the behavior of elders toward water usage has been difficult [39], mainly because of the lack of available information on appropriate water consumption practices and unwillingness to change traditional habits; these factors may explain the low usage of washing machines and solar water heaters in the study area. Households with more family members used larger quantities of water, but the average water consumption per person decreased. Martin [40] and Keshavarzi et al. [14] showed that water consumption per capita is lower in large families than in small families because some water usage activities (use for kitchen, vegetable gardening, livestock needs, and house and yard cleaning) are relatively independent of family size,–findings that the current work verifies. Rural domestic water consumption in the Wei River Basin increased from 37.3 Lpcd in 1999 [41] to present-day 56.2 Lpcd–a rise that can be explained by the decline in net family size from 4.2 in 1989 to 3.0 in 2010.

Determining the factors that affect domestic water consumption is difficult because of the complexities of water use patterns (when, how, and why particular water use activities take place in a day); these patterns are, in turn, influenced by numerous factors, as discussed in Krantz [42]. The attempts to determine the key factors effect on water consumption showed that the explanatory power of the factors toward water consumption is modest more often than not [11], with R^2^ values of no more than 0.40; from case studies in Mexico (R^2^ = 0.13) [10], Syme et al.’s [9] study in Perth, Australia (R^2^ = 0.22), and the current work (R^2^ = 0.37) (Table 6). The low explanatory power of the factors (R^2^ values) in these studies suggest that there are much more underlying factors impacting on water use that are not yet to be discovered.

Water prices have been significantly associated with water consumption [43], [44], but the studies that report such finding are those on the large water consumption of households and the concerns of users regarding high water prices [45]. In rural areas of developing countries, the total water consumption per capita per day is usually less than 50 L [14], [15]. Our study found that the average amount in rural Wei River Basin was approximately 56.2 Lpcd, of which indoor use and vegetable gardening accounted for the majority of consumption. Given the importance of vegetable gardening for rural families [38], more than 90% of the residents were unwilling to minimize this consumption practice, regardless of current water prices, which is US$ 0.16–0.32/m^3^ compared with US$ 4580–4810 annual household income (Table1). Poor management of non-metered piped water supply and low price transparency (bundling with electric bills) were the other reasons why water price was not significantly associated with water consumption in the rural areas studied.

As revealed by Syme et al. [9] and Loh and Coghlan [46], domestic water consumption is significantly associated with the living standards of consumers and the use of water appliances. In the Wei River Basin, the increase in Lpcd in the recent decade can be partly explained by the use of solar water heaters and washing machines. However, the present utilization rate for washing machines remains very low, even in the villages with continuous water supply, in which the utilization rate for washing machines is less than 0.20 (Table 3). This result is attributed to traditional practices and the lack of knowledge of elders regarding water usages. Although the living standards of residents and water supply facilities in rural areas of developing countries have significantly improved in recent decades, the present findings raise concerns on traditional lifestyles and water usages; specifically, concerns exist as to the continued important role of such lifestyles and usages in rural domestic water consumption after the implementation of improved water supply systems (Table 3). Moreover, a large, temporary, and seasonal rural-to-urban migration of young people in developing countries occurs, with approximately 200 million and 120 million migrants per year in India and in China, respectively [47]. This migration means elder family members are left behind in rural areas. This phenomenon and the characteristics of water consumers, particularly elders (e.g., education, water availability, and preference for vegetable gardening and livestock rearing, traditional water consumption practices), should be considered in research on the factors that affect domestic water consumption and water use behavior.

### Conclusion

Domestic water consumption in rural China is highly affected by water supply patterns, the characteristics of heads of households, vegetable gardening, and the use of water appliances. Despite considerably improved water supply facilities and living standards in rural areas over the past two decades, traditional lifestyles and household habits (low use of water appliances and preference for vegetable gardening) continue to significantly affect domestic water consumption. We do not recommend public tap access, intermittent water supply, and the use of price mechanisms as variables for consideration in water management for rural regions because the effect of such variables will be negated by high sanitation risks, the importance of vegetable gardens to households, and poor water management methods (e.g., non-metered piped water supply, poor water pricing transparency). The traditional habits and cultural backgrounds of water consumers should be thoroughly examined in formulating water management schemes for rural areas.
